# Fluorinated groups mediate the immunomodulatory effects of volatile anesthetics in acute pulmonary inflammation

**DOI:** 10.1186/cc9839

**Published:** 2011-03-11

**Authors:** M Urner, I Herrmann, B Müller-Edenborn, L Limbach, W Stark, B Beck-Schimmer

**Affiliations:** 1 University Hospital Zurich, Switzerland; 2 ETH Zurich, Switzerland

## Introduction

Volatile anaesthetics are known as immunomodulatory substances in inflammatory as well as in ischemia/reperfusion processes [1,2]. We investigated in a model of acute pulmonary inflammation whether these immunomodulatory effects arise from the ether basic structure or from characteristics in their halogenation.

## Methods

Inflammatory response in pulmonary epithelial and endo-thelial cells as well as in neutrophils after co-exposure to endotoxin and sevoflurane, diethyl-ether or various water-soluble molecules carrying trifluorinated carbon groups (CF3) was evaluated. Expression of monocyte chemotactic protein-1 and cytokine-induced neutrophil chemoattractant protein-1, IL-6, and IL-8 as a measure of inflammatory activity were analyzed by ELISA. Chemotactic activity of supernatants regarding neutrophil recruitment was assessed. Flow cytometric analysis of neutrophil activation was performed measuring CD11b and CD62L expression. Viability was observed using fluorescence DNA quantitation. Cytotoxicity was evaluated by measuring lactate dehydrogenase in supernatants.

## Results

Expression of inflammatory mediators to lipopolysaccharide stimulation in epithelial and endothelial cells was dose-dependently decreased upon exposure to sevoflurane and other molecules with CF3 groups. This was not observed for diethyl-ether or structure-similar nonfluorinated molecules. In neutrophils, chemotactic activity as well as expression of surface CD11b and CD62L was decreased by molecules carrying CF3 groups. Cytotoxicity could be excluded.

## Conclusions

These findings show that the immunomodulatory effects are not limited to volatile anesthetics, but are associated with a much broader class of CF3 group-containing molecules. The immunomodulatory effects could now be provided in a hydrophilic, injectable formulation for the future treatment of patients suffering from acute pulmonary inflammation in environments not suitable for volatile anesthetics.
